# Supplementary material for: Identification of photoperiod-induced specific miRNAs in the adrenal glands of Sunite sheep (Ovis aries)
Source: Front Vet Sci. 2022 Jul 22;9:888207. doi: 10.3389/fvets.2022.888207 (PMC9354845; doi:10.3389/fvets.2022.888207)
Supplement: Supplementary file 1 [file Data_Sheet_1.ZIP › Supplementary file/miRDeep2 file.zip!/miRDeep2 file/Know_Structure/SPLP42A3/oar-mir-30a.pdf]

miRBase precursor : oar-mir-30a
Total read count : 51342
oar-miR-30a-5p read count : 44469
oar-miR-30a-3p read count : 6873
remaining reads : 0

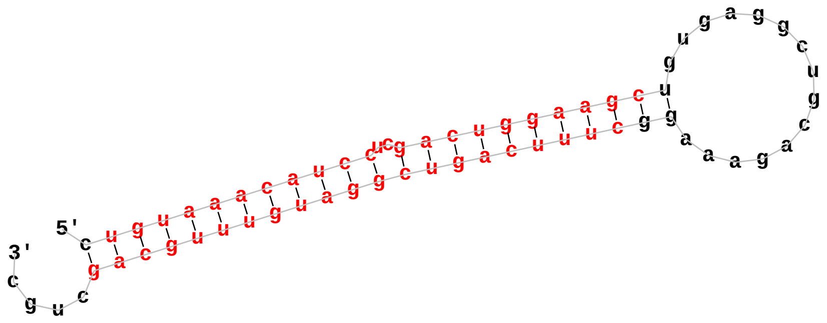

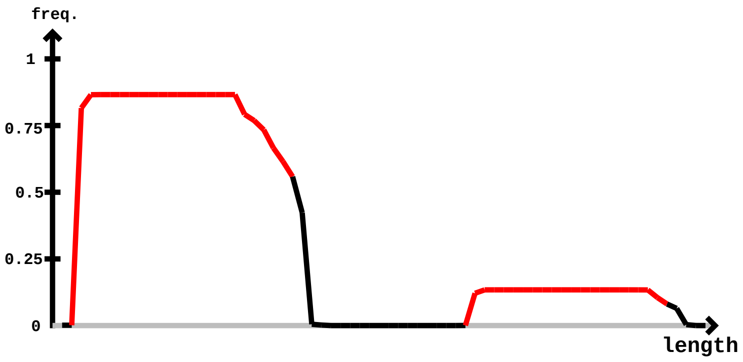

oar-miR-30a-3p

oar-miR-30a-5p

5'- cuguaaacauccucgacuggaagcugugaggcugcagaaaggcuuucagucggauguuugcagcugc -3' exp

((((((((((((..(((((((((((..............))))))))))))))))))))))))....	reads	mm	sample
cuguaaacauccuUgacu..	4	1	seq
cuguaaacauccucgacu..	2	0	seq
cuguaaacauccucgacug...	2	0	seq
cuguaaacauccucgacugg..	1	0	seq
cuguaaacauccuUgacugg..	2	1	seq
cuguaaacauccucgacugga...	11	0	seq
cuguaaacauccucgacuggaa..	7	0	seq
cuguaaacauccucgacuggaag...	6	0	seq
cuguaaacauccuUgacuggaagc..	2	1	seq
cuguaaacauccucgacuggaagc..	15	0	seq
cuguaaacauccucgacuggaagcu...	10	0	seq
cuguaaacauccuUgacuggaagcu...	6	1	seq
.uguaaacauccucgacu..	448	0	seq
.uguaaacauccuUgacu..	3351	1	seq
.uguaaacauccuUgacug...	829	1	seq
.uguaaacauccucgacug...	363	0	seq
.uguaaacauccucgacugg..	784	0	seq
.uguaaacauccuUgacugg..	1003	1	seq
.uguaaacauccucgacugga...	3002	0	seq
.uguaaacauccuUgacugga...	452	1	seq
.uguaaacauccuUgacuggaa..	329	1	seq
.uguaaacauccucgacuggaa..	2268	0	seq
.uguaaacauccucgacuggaag...	2052	0	seq
.uguaaacauccuUgacuggaag...	848	1	seq
.uguaaacauccucgacuggaagc..	5909	0	seq
.uguaaacauccuUgacuggaagc..	960	1	seq
.uguaaacauccuUgacuggaagcu...	11764	1	seq
.uguaaacauccucgacuggaagcu...	7411	0	seq
.uguaaacauccuUgacuggaagcug..	29	1	seq
.uguaaacauccucgacuggaagcug..	1	0	seq
.uguaaacauccuUgacuggaagcugu.......................................	6	1	seq
.uguaaacauccucgacuggaagcugu.......................................	1	0	seq
..guaaacauccuUgacug...	1	1	seq
..guaaacauccucgacugg..	1	0	seq

```
                                         oar-miR-30a-3p
 oar-miR-30a-5p
cuguaaacauccucgacuggaagcugugaggcugcagaaaggcuuucagucggauguuugcagcugc

..guaaacauccuUgacugga..............................................    3      1      seq
..guaaacauccucgacugga..............................................    4      0      seq
..guaaacauccuUgacuggaa.............................................    5      1      seq
..guaaacauccucgacuggaa.............................................    4      0      seq
..guaaacauccucgacuggaag............................................    4      0      seq
..guaaacauccuUgacuggaag............................................    12     1      seq
..guaaacauccuUgacuggaagc...........................................    26     1      seq
..guaaacauccucgacuggaagc...........................................    16     0      seq
..guaaacauccucgacuggaagcu..........................................    95     0      seq
..guaaacauccuUgacuggaagcu..........................................    2189   1      seq
..guaaacauccucgacuggaagcug.........................................    2      0      seq
..guaaacauccuUgacuggaagcug.........................................    117    1      seq
..guaaacauccuUgacuggaagcugu........................................    90     1      seq
...uaaacauccucgacuggaagc...........................................    1      0      seq
...uaaacauccucgacuggaagcu..........................................    5      0      seq
...uaaacauccuUgacuggaagcu..........................................    4      1      seq
...uaaacauccuUgacuggaagcug.........................................    4      1      seq
...uaaacauccuUgacuggaagcugu........................................    5      1      seq
....aaacauccuUgacuggaagcu..........................................    1      1      seq
....aaacauccuUgacuggaagcug.........................................    1      1      seq
....aaacauccuUgacuggaagcugu........................................    1      1      seq
.........................................gcuuucagucggauguuuAc......    1      1      seq
.........................................gcuuucagucggauguuuAca.....    11     1      seq
.........................................gcuuucagucggauguuuAcag....    1      1      seq
.........................................gcuuucagucggauguuuAcagc...    8      1      seq
..........................................cuuucagucggauguuu........    6      0      seq
..........................................cuuucagucggauguuug.......    2      0      seq
..........................................cuuucagucggauguuuA.......    7      1      seq
..........................................cuuucagucggauguuuAc......    1168   1      seq
..........................................cuuucagucggauguuugc......    188    0      seq
..........................................cuuucagucggauguuuAca.....    1064   1      seq
..........................................cuuucagucggauguuugca.....    117    0      seq
..........................................cuuucagucggauguuugcag....    127    0      seq
..........................................cuuucagucggauguuuAcag....    671    1      seq
..........................................cuuucagucggauguuugcagc...    577    0      seq
..........................................cuuucagucggauguuuAcagc...    2232   1      seq
..........................................cuuucagucggauguuugcagcu..    40     0      seq
..........................................cuuucagucggauguuugcagcug.    1      0      seq
...........................................uuucagucggauguuugc......    28     0      seq
...........................................uuucagucggauguuuAc......    90     1      seq
...........................................uuucagucggauguuuAca.....    36     1      seq
...........................................uuucagucggauguuugca.....    3      0      seq
...........................................uuucagucggauguuugcag....    17     0      seq
...........................................uuucagucggauguuuAcag....    29     1      seq
...........................................uuucagucggauguuuAcagc...    187    1      seq
...........................................uuucagucggauguuugcagc...    111    0      seq
...........................................uuucagucggauguuugcagcu..    143    0      seq
...........................................uuucagucggauguuugcagcug.    2      0      seq
............................................uucagucggauguuugc......    1      0      seq
............................................uucagucggauguuuAc......    1      1      seq
............................................uucagucggauguuugcag....    1      0      seq
............................................uucagucggauguuuAcagc...    2      1      seq
............................................uucagucggauguuugcagcu..    1      0      seq
```
